# N6-methyladenosine-related Genomic Targets are Altered in Breast Cancer Tissue and Associated with Poor Survival

**DOI:** 10.7150/jca.35053

**Published:** 2019-08-29

**Authors:** Liwen Liu, Xin Liu, Zihui Dong, Jianhao Li, Yan Yu, Xiaolong Chen, Fang Ren, Guangying Cui, Ranran Sun

**Affiliations:** 1 Precision Medicine Center, The First Affiliated Hospital of Zhengzhou University, Zhengzhou 450052, China; 2 Key Laboratory of Clinical Medicine, The First Affiliated Hospital of Zhengzhou University, Zhengzhou 450052, China; 3 National Engineering Laboratory for Internet Medical System and Application, The First Affiliated Hospital of Zhengzhou University, Zhengzhou 450052, Henan, China

**Keywords:** N6-methyladenosine, Breast cancer, YTHDF3, Prognosis, Biomarkers.

## Abstract

**Purpose**: The ectopic expression of N6-methyladenosine (m6A) associated genes is a common feature of multiple tumors. However, little is known about the expression status and the prognostic value of these genes in human breast cancer (BRC). Herein, we conducted a comprehensive analysis to identify the expression profiling and clinical significance of m6A-related genomic targets in BRC.

**Materials and Methods**: The expression data including 1109 BRC tissues and 113 normal breast tissues were obtained from The Cancer Genome Atlas (TCGA) database to evaluate the mRNA expression levels of m6A-related genomic targets. In addition, 6 independent BRCA cohorts retrieved from the Gene Expression Omnibus (GEO) database were enrolled to further ascertain the expression profiling of m6A-related genomic targets. Meanwhile, the immunohistochemical (IHC) staining data from BRC tissue microarray (TMA) cohort and the Human Protein Atlas (HPA) database were used to evaluate the proteomic expression of m6A-related genomic targets. Immunofluorescence (IF) analysis was performed to validate the subcellular location of m6A-related genomic targets. Moreover, the prognostic value of m6A-related genomic targets in BRC was analyzed by Kaplan-Meier analysis and Cox regression models.

**Results**: m6A-related genomic targets were differentially expressed in BRC tissues. TMA IHC staining showed that most of the m6A-related genomic targets were significantly altered at the protein level (either upregulated or downregulated), consistent with their changes in the genomic profile. IF analysis showed the subcellular location of m6A-related genomic targets in BRC cell lines. Furthermore, we demonstrated that overexpression of YTHDF1 (P=0.049), YTHDF3 (P<0.001) and KIAA1429 (P=0.032) predicted poor prognosis in terms of overall survival (OS). Upregulation of YTHDF3 was an independent prognostic factor for OS in patients with BRC (P=0.036).

**Conclusion**: m6A-related genomic targets are significantly altered in BRC and predict poor prognosis. These m6A-related genomic targets could serve as novel prognostic biomarkers for BRC.

## Introduction

Breast cancer (BRC) is the most prevalent malignancy with a heterogeneous group of molecular subtypes 1. BRC is also the leading cause of cancer-related death for women in the vast majority countries, with an estimated 2.1 million newly diagnosed BRC cases and 626 thousands deaths predicted in 2018, accounting for almost 25% cancer cases and 6.6% cancer deaths among women worldwide 2, 3. Despite a number of available treatment strategies, the prognosis of BRC remains poor. Hence, identifying additional genomic targets for the early diagnosis and effective treatment of BRC is an ever-increasing need.

Epigenetic dysregulation is a consistent feature in multiple cancers 4. In addition to the well-known classical epigenetic modulating mechanisms, such as chromatin remodelling 5, DNA methylation 6 and histone modifications 7, RNA N6-methyladenosine (m6A) has an emerging recognized role as an epigenetic regulator. m6A, a prevalent internal modification in mammalian mRNAs and noncoding RNAs, has emerged in a ubiquitous role to fine tune RNA processing by acting as “writers”, “erasers” and “readers” 8. Through this special approach, m6A provides a new mechanism of epigenetic regulation in many bioprocesses such as meiosis 9, sex determination 10, neural development 11, cardiac rhythms 12 and stress response 13. Interestingly, m6A is also required for some human diseases, and recent publications in the cancer research field have provided new insights into m6A. Multiple studies have identified that several m6A-related molecules are involved in mutagenesis and carcinogenesis 14-16. However, little is known about the critical role of m6A-related genomic targets in BRC.

In this study, we identified a class of m6A-related genomic targets expression profiles in BRC based on The Cancer Genome Atlas (TCGA) and the Gene Expression Omnibus database (GEO). In addition, immunofluorescence (IF) staining and tissue microarray (TMA) analysis were performed to detect subcellular locations and expression patterns of m6A-related genomic targets at the protein level. Furthermore, we also evaluated the robustness of m6A-related genomic targets dysregulation in patients through survival analysis to explore the potential value of m6A in BRC. Our findings suggest that m6A-related genomic targets could serve as novel prognostic biomarkers for BRC.

## Materials and Methods

### Expression data sets

The TCGA-BRCA cohort data of 1109 BRC patients and 113 normal patients were downloaded from The Cancer Genome Atlas (TCGA, https://tcga-data.nci.nih.gov/tcga/). 6 independent cohorts, including GSE70947, GSE15852, GSE109169, GSE36295, GSE29044 and GSE24124, were obtained from the Gene Expression Omnibus (GEO https://www.ncbi.nlm.nih.gov/geo/) database. These datasets were used to analyse the expression profiles of m6A-associated molecules in BRC and evaluate their correlations with the clinical prognosis.

### Cell lines and cell culture

Human BRC cell lines MDA-MB-231, MDA-MB-468 and MCF-7 were purchased from ATCC (Manassas, USA). Cells were maintained at 37°C in a humidified atmosphere of 5% CO_2_ in Dulbecco's modified Eagle medium (DMEM) supplemented with 10% fetal bovine serum (Gibco, New York, NY, USA) and 100 U/mL penicillin/streptomycin (Corning, New York, NY, USA).

### TMA cohorts

The tissue microarray (TMA) containing 20 BRC specimens and 20 normal breast tissue specimens was obtained from the First Affiliated Hospital of Zhengzhou University. The Institutional Review Board of the First Affiliated Hospital of Zhengzhou University approved this study. And informed consent for this study was collected from all participants. To further confirm the expression of m6A-related genes, we analysed another BRC TMA cohort obtained from the Human Protein Atlas (HPA, https://www.proteinatlas.org/) database.

### Immunohistochemical staining

TMA sections (5 μm) were deparaffinized, hydrated, blocked for endogenous peroxidases and antigen retrieval. After blocking for an hour at room temperature, the slide was incubated with primary antibody at 4°C overnight. And then samples were probed with biotinylated goat anti-rabbit secondary antibody. Finally, the slide was detected by SignalStain® DAB (Cell Signaling Technology, Danvers, MA) and counterstained with haematoxylin QS (Vector Laboratories). Cells containing brown granules were independently counted by two pathologists who were blinded to clinical parameters, and the samples were scored according to the proportion of positive cells as follows: 0, none; 1, <25%; 2, 25%-50%; 3, 51%-75%; and 4, 76%-100%. The staining intensity was scored as follows: 0, none; 1, weak; 2, moderate; and 3, strong. The total staining score (range 0-12) was calculated by multiplying the two subscores, and the samples with scores of 0-3, 4-6 and 9-12 were respectively classified as low expression, moderate expression and high expression.

### Immunofluorescence assay

For immunofluorescence (IF) analysis, the BRC cells cultured in 24-well plate were fixed with 4% paraformaldehyde (PFA) in PBS for 20 min at room temperature. And then cells were permeabilized with 0.5% Triton X-100 (Solarbio, Shanghai, China) in PBS. After blocking with 1% bovine serum albumin (BSA), we incubated the cells with primary antibody at 4°C overnight and then the appropriate corresponding secondary antibodies (Jackson ImmunoResearch Inc., USA) at room temperature for 30 min. Finally, the nuclei were counterstained with DAPI (Beyotime, China), and images were obtained with a Zeiss Axio microscope (Zeiss, Oberkochen, Germany). The detailed information of antibodies used in this study is listed in table S.

### Statistical analysis

Statistical analysis was performed using SPSS software (version 23.0, Inc., Chicago, IL) and GraphPad Prism 6 software (GraphPad Software, Inc., La Jolla, CA, USA). The differences between two independent groups were analysed using Student's *t* test (unpaired, two-tailed). For each significantly ectopically expressed molecule, a Kaplan-Meier overall survival and relapse-free survival analysis were performed with a log-rank test. Cox regression analysis of univariate and multivariate was performed to ascertain independent factors. A P<0.05 was considered statistically significant.

## Results

### Distinct differential expression profiles of m6A-related genomic targets in BRC

**Figure 1:**
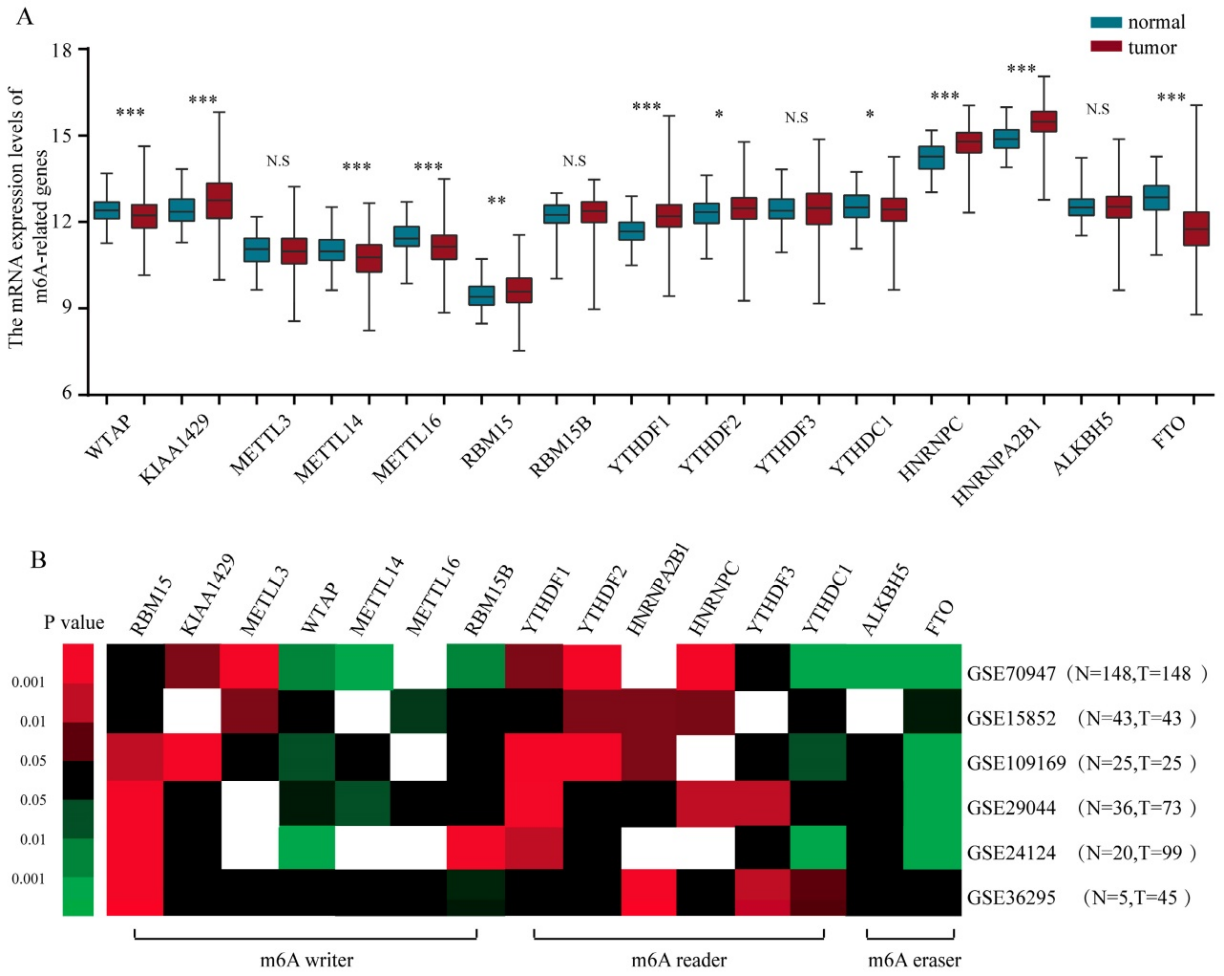
** The altered expression profiles of m6A related genes in human BRC tissues. (A)** The mRNA expression profiles of m6A-related genes in TCGA-BRCA cohort. **(B)** Heatmap showing the mRNA expression alteration of m6A related genomic targets in six independent GEO microarray datasets. Red means up-regulated; green means down-regulated; black means not significant; blank means genes are not expressed or absent in the datasets. Statistical analysis was performed in Student's *t* test (unpaired, two-tailed)

To gain a holistic insight into the m6A-related genomic aberrations underlying BRC, we downloaded RNA transcriptomic datasets containing next generation sequencing (RNA-seq) data of 1109 BRC tissues and 113 non-tumor tissues from TCGA project (TCGA-BRCA). We analyzed the mRNA expression levels of the known m6A-related genomic targets including m6A “writers”, such as WTAP (Wilms' tumour 1-associated protein), RBM15, KIAA1429, METTL3, METTL14, METTL16 and RBM15B, m6A “readers”, such as YTHDF1, YTHDF2, YTHDF3, YTHDC1, HNRNPC and HNRNPA2B1, and m6A “erasers”, such as FTO and ALKBH5 (**Figure 1A**). The results showed that a considerable number of m6A-related genomic targets were differentially expressed in BRC tissues in comparison with those in normal breast tissues (**Figure 1A**). Of these genes, 6 genes such as KIAA1429 (P<0.001), RBM15 (P<0.01), YTHDF1 (P<0.001), YTHDF2 (P=0.022), HNRNPC (P<0.001) and HNRNPA2B1 (P<0.001) were upregulated and 5 genes such as WTAP (P<0.001), METTL14 (P<0.001), METTL16 (P<0.001), YTHDC1 (P=0.013) and FTO (P<0.001) were downregulated in BRC tissues, while others were not significantly different. Furthermore, we validated the expression of these aberrant m6A-associated genomic targets in 6 independent BRCA GEO datasets with microarray platforms (**Table 1**). Notably, GEO dataset analysis showed that the m6A-related genomic targets exhibited similar expression patterns in BRC (**Figure 1B**). Taken together, these data indicate a strong deregulation of several m6A-related genomic targets in human BRC and show that these alterations are broadly consistent across clinical cohorts.

### Altered m6A-related genomic targets show similar expression patterns at the protein level

**Figure 2:**
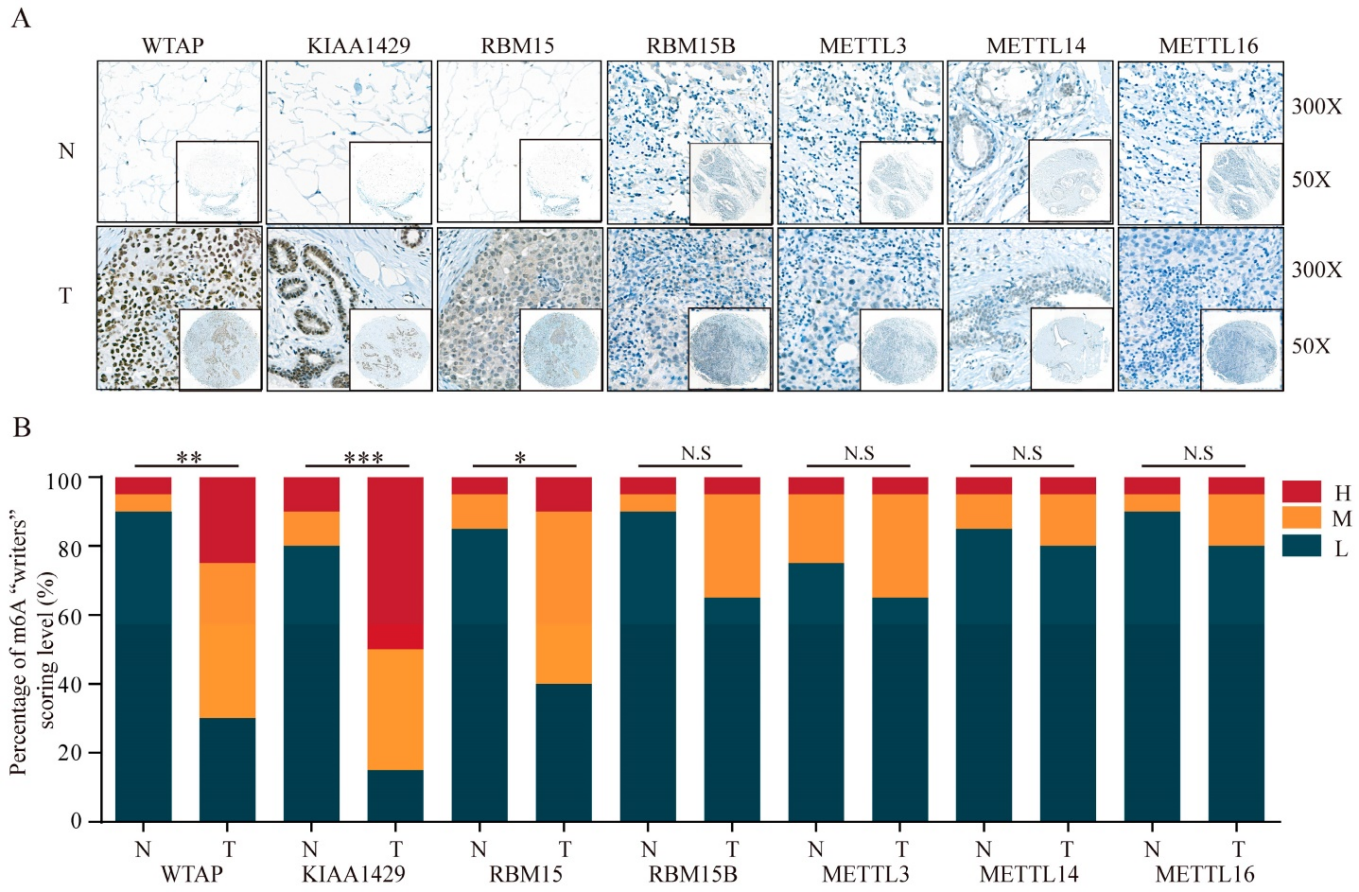
** The protein levels of m6A “writers” are differentially expressed in BRC tissues and adjacent normal tissues. (A)** Representative IHC staining result of m6A “writers” in BRC tissues (T) and the adjacent normal tissues (N) was shown. **(B)** The distribution of differentially expressed m6A “writers” in normal breast tissues (N) and breast cancer tissues (T) was quantified. L, low expression; M, moderate expression; H, high expression; N.S, not significant; *P < 0.05; **P < 0.01; ***P< 0.001.

**Figure 3:**
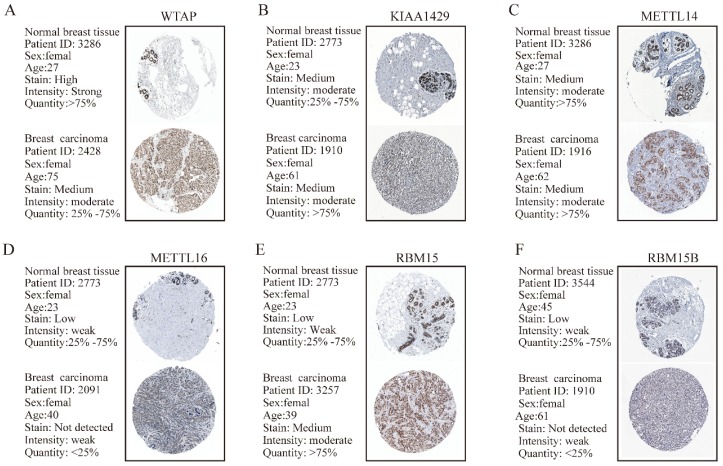
** The protein expression levels of M6A “writers” in breast carcinoma tissues and normal breast tissues from HPA database are determined by IHC staining. (A)** Representative IHC staining of WTAP in breast carcinoma and normal breast tissues. **(B)** Representative IHC staining of KIAA1429 in breast carcinoma and normal breast tissues. **(C)** Representative IHC staining of METTL14 in breast carcinoma and normal breast tissues. **(D)** Representative IHC staining of METTL16 in breast carcinoma and normal breast tissues. **(E)** Representative IHC staining of RBM15 in breast carcinoma and normal breast tissues. **(F)** Representative IHC staining of RBM15B in breast carcinoma and normal breast tissues.

TMA data from the ZZU cohort and the Human Protein Atlas (HPA) database were further analyzed to validate the expression patterns of altered m6A-related genomic targets in BRC tissues and noncancerous breast tissues at the protein level. First, we examined the expression levels of m6A “writers” in BRC tissue microarrays comprising 20 BRC specimens and adjacent normal specimens. Immunohistochemistry (IHC) staining analysis indicated the significant upregulation of WTAP (P=0.002), KIAA1429 (P<0.001) and RBM15 (P=0.012) in BRC specimens (**Figure 2A and 2B**). While the corresponding mRNA expression level of WTAP was lower in BRC tissues, the protein expression levels of KIAA1429 and RBM15 were consistent with their transcriptional levels in comparison with those in the adjacent normal tissues. In contrast, we did not detect any significant changes in protein expression levels of METTL3, METTL14, METTL16 or RBM15B between BRC and adjacent normal tissues (**Figure 2A** and** 2B**). Moreover, IHC staining results obtained from the HPA database also demonstrated the similar expression patterns of m6A “writers” in BRC, consistent with their mRNA levels (**Figure 3A-F**). Altogether, these results further confirmed the highly significant dysregulation of m6A “writers” in BRC.

**Figure 4:**
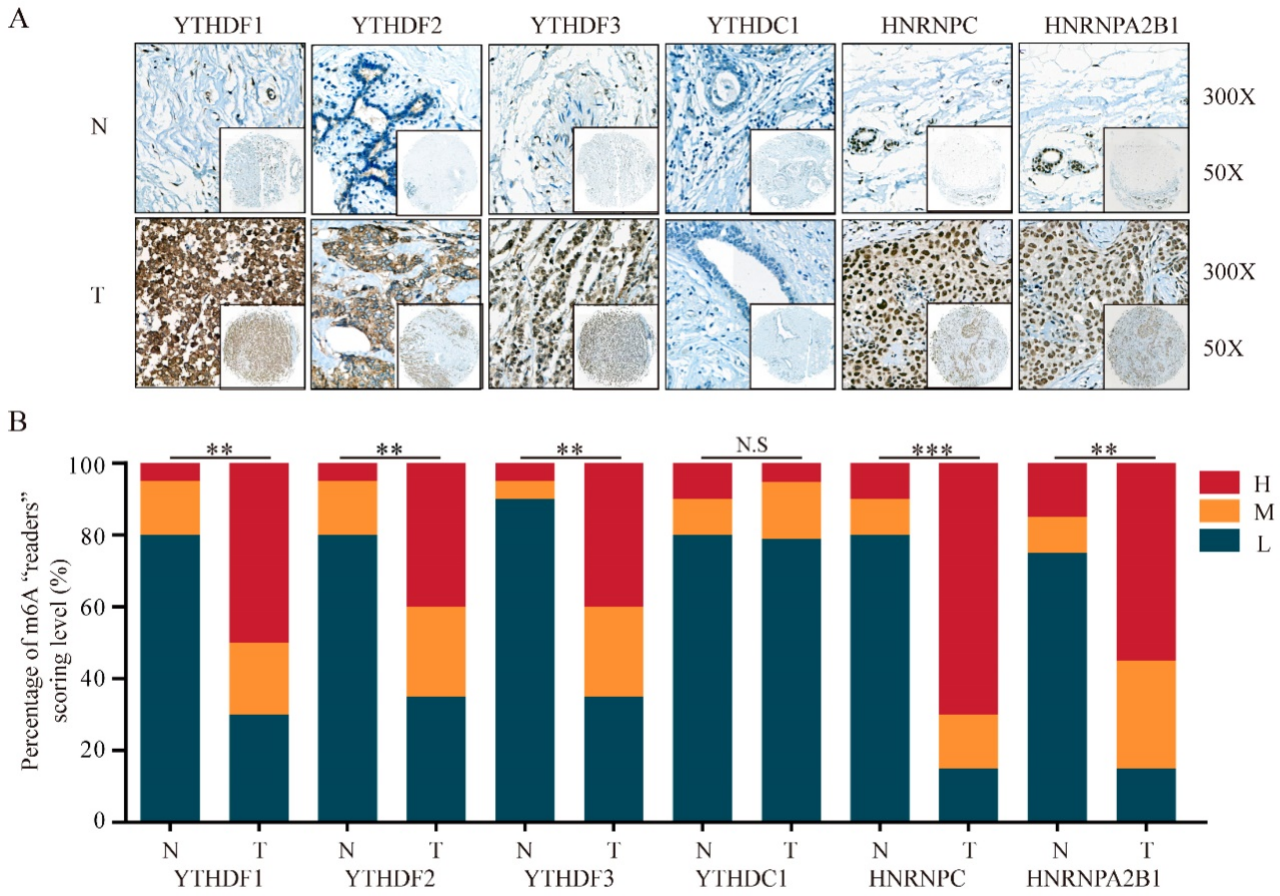
** The protein levels of m6A “readers” are differentially expressed in BRC tissues and adjacent normal tissues. (A)** Representative IHC staining result of m6A “readers” in BRC tissues (T) and the adjacent normal tissues (N) was shown. **(B)** The distribution of differentially expressed m6A “readers” in normal breast tissues (N) and breast cancer tissues (T) was quantified. L, low expression; M, moderate expression; H, high expression; N.S, not significant; **P < 0.01; ***P< 0.001.

**Figure 5:**
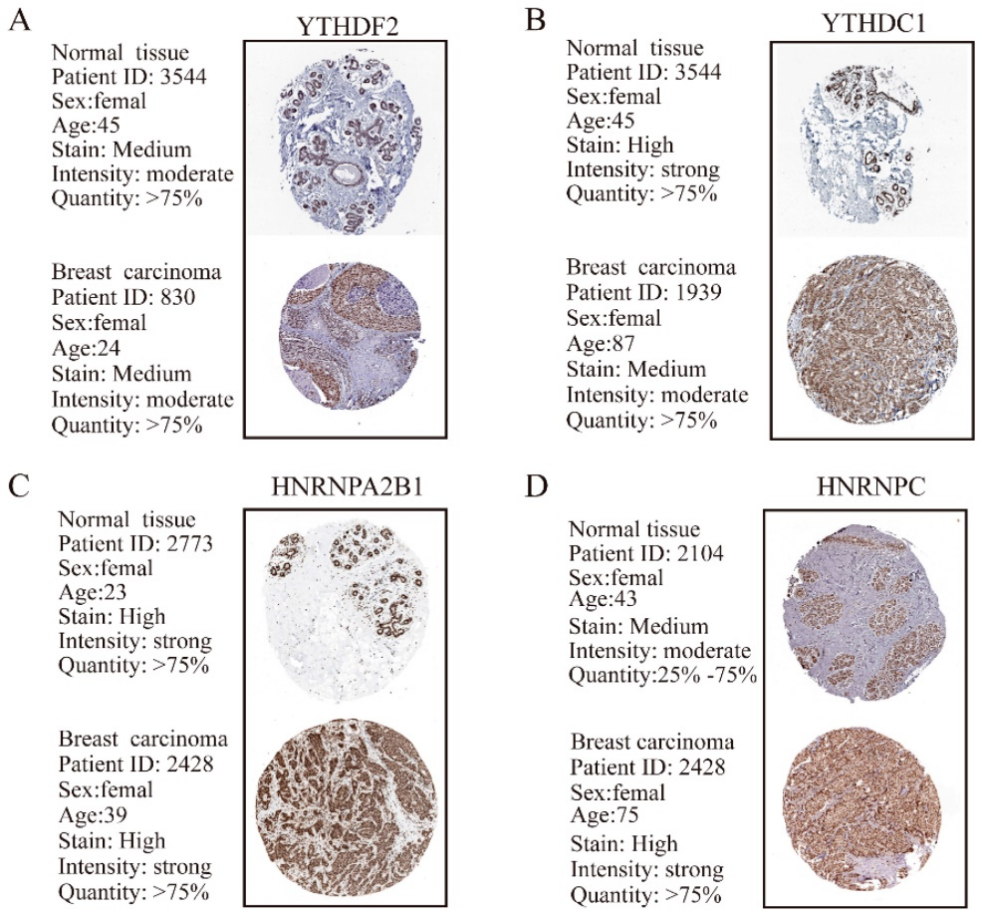
** The protein expression levels of M6A “readers” in breast carcinoma tissues and normal breast tissues from HPA database are determined by IHC staining. (A)** Representative IHC staining of YTHDF2 in breast carcinoma and normal breast tissues. **(B)** Representative IHC staining of YTHDC1 in breast carcinoma and normal breast tissues. **(C)** Representative IHC staining of HNRNPA2B1 in breast carcinoma and normal breast tissues. **(D)** Representative IHC staining of HNRNPC in breast carcinoma and normal breast tissues.

In addition to m6A “writers”, we also analyzed the expression of m6A “readers” in BRC tissues. IHC staining results showed that the majority of genes associated with m6A “readers” displayed markedly differential expression in BRC TMA. While the expression of YTHDC1 showed no significant difference, m6A “readers” including YTHDF1 (P=0.002), YTHDF2 (P=0.009), YTHDF3 (P=0.002), HNRNPC (P<0.001) and HNRNPA2B1 (P=0.001) were significantly overexpressed in BRC tissues, consistent with the changes of their mRNA expression levels (**Figure 4A** and **4B**). Furthermore, HPA analysis showed that YTHDC1 and HNRNPC were highly expressed in a pattern similar to the mRNA level changes in BRC tissues (**Figure 5B** and** 5D**). However, YTHDF2 and HNRNPA2B1 did not show prominent changes at the protein levels between BRC tissues and normal breast tissues (**Figure 5A** and **5C**). The heterogeneity between the HPA data and our TMA cohort's results may be ascribed to the differences in test platforms. Moreover, YTHDF1 and YTHDF3 were absent from the HPA database. The results confirm m6A “readers” as highly altered molecules in BRC, indicating large variations in the expression of these genomic targets in BRC patients.

**Figure 6:**
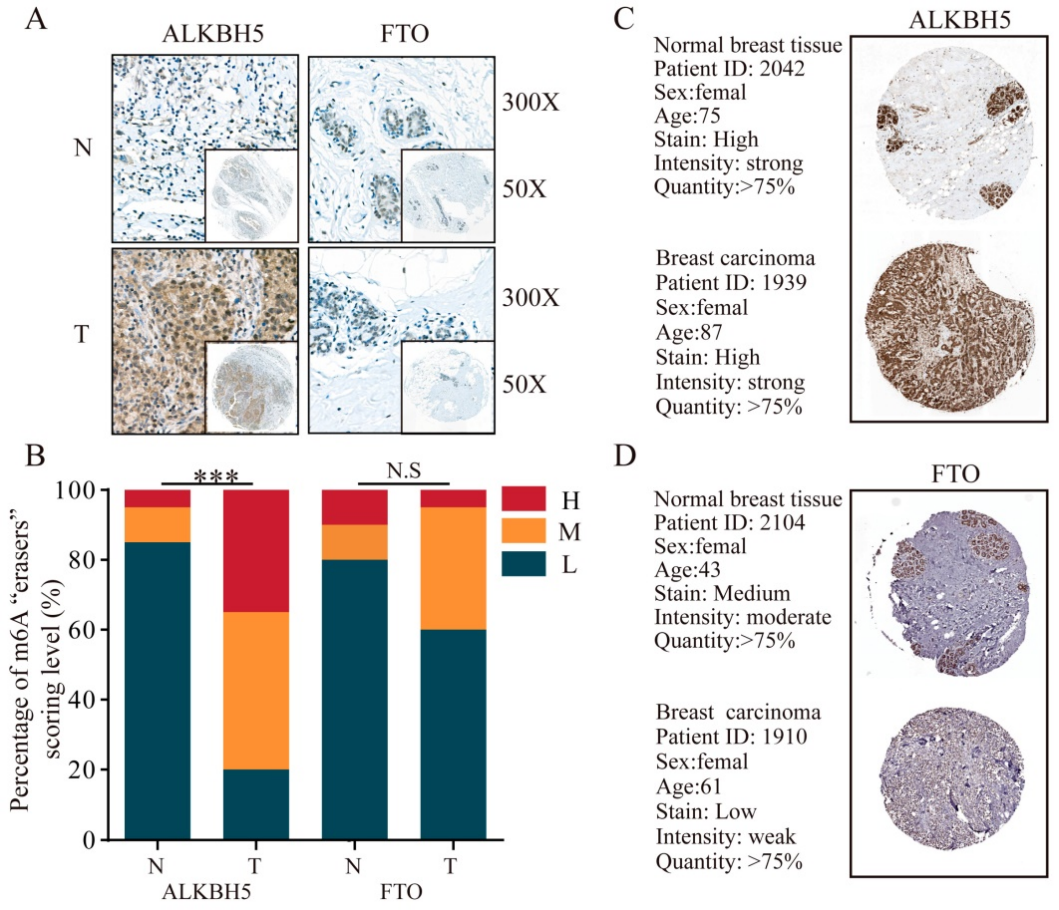
** The protein levels of m6A “erasers” are differentially expressed in BRC tissues and adjacent normal tissues. (A)** Representative IHC staining result of m6A “erasers” in BRC tissues (T) and the adjacent normal tissues (N) was shown. **(B)** The distribution of differentially expressed m6A “erasers” in normal breast tissues (N) and breast cancer tissues (T) was quantified.** (C)** Representative IHC staining of ALKBH5 in breast carcinoma and normal breast tissues from HPA database.** (D)** Representative IHC staining of FTO in breast carcinoma and normal breast tissues from HPA database. L, low expression; M, moderate expression; H, high expression; N.S, not significant. ***P< 0.001.

Furthermore, for the only two known m6A “eraser” demethylases, FTO and ALKBH5, we detected a much stronger expression of ALKBH5 (P<0.001) in BRC tissues compared with that in normal breast tissues, whereas FTO had no obvious changes (**Figure 6A** and **6B**). IHC staining data from HPA database showed a predominant upregulation of ALKBH5 but downregulation of FTO in breast carcinoma tissues in comparison with normal breast tissues (**Figure 6C** and **6D**).

### Subcellular location of m6A-associated genomic targets in BRC cell lines

**Figure 7:**
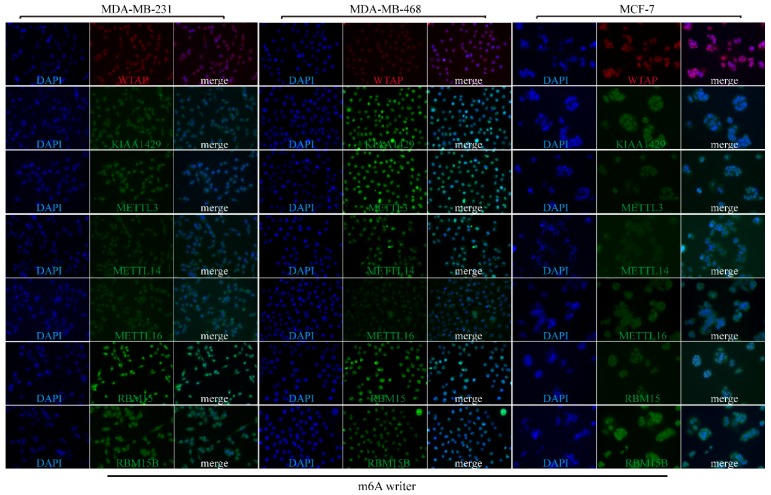
** Subcellular location of m6A “writers” in three different BRC cell lines.** MDA-MB-231, MDA-MB-468 and MCF-7 cells were stained by DAPI and different m6A “writers” and representative immunofluorescence photographs of m6A “writers” subcellular location in MDA-MB-231, MDA-MB-468 and MCF-7 cells were shown.

**Figure 8:**
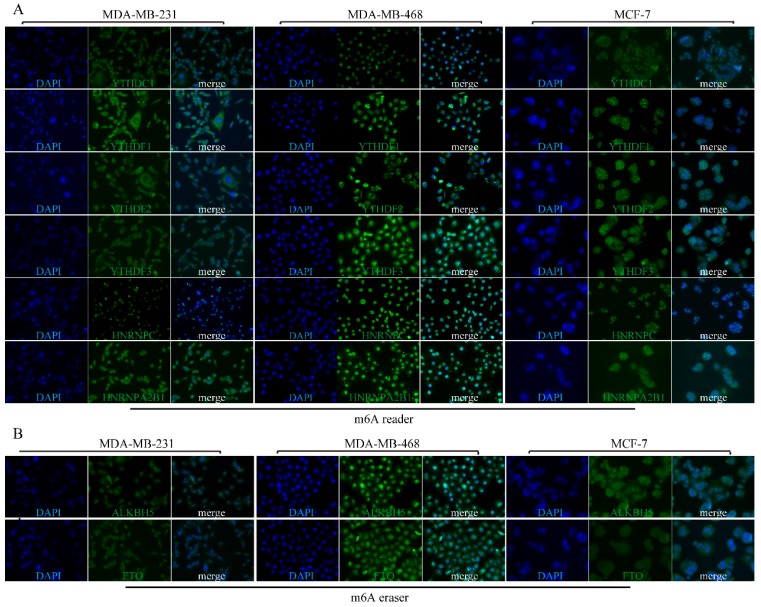
** Subcellular location of m6A “readers” and “erasers” in three different BRC cell lines.** MDA-MB-231, MDA-MB-468 and MCF-7 cells were stained by DAPI and different m6A “readers” **(A)** or “erasers” **(B)** and representative immunofluorescence photographs of m6A “reader” or “erasers” subcellular location in MDA-MB-231, MDA-MB-468 and MCF-7 cells were shown.

To explore the subcellular location of m6A-related genomic targets in BRC cell lines and their potential regulation mechanisms, immunofluorescence (IF) assay was performed on three different BRC cell lines (MDA-MB-231, MDA-MB-468 and MCF-7). As shown in **Figure 7**, there was strong nuclear staining as well as weak cytoplasmic staining for most of the m6A “writers” (WTAP, KIAA1429, METTL13, RBM15, and RBM15B) in the three BRC cell lines. However, the fluorescence signals of METTL14 and METTL16 in MDA-MB-231, MDA-MB-468 and MCF-7 were not significantly enhanced. For m6A “readers”, IF staining showed that YTHDC1 was mainly located in the nucleus especially in MDA-MB-468. On the contrary, YTHDF1 and YTHDF2 were mainly located in the cytoplasm, while HNRNPC was only detected in the nucleus (**Figure 8A**). YTHDF3 and HNRNPA2B1 were expressed both in the cytoplasm and nucleus (**Figure 8A).** We also detected fluorescence signals for ALKBH5 and FTO both in the cytoplasm and nucleus (**Figure 8B**). Furthermore, the expression intensity of FTO was weak in BRC cell lines, especially in MCF-7. These findings were similar to the IHC staining results in BRC tissues. The special subcellular location may further hint at a potential dynamic regulation of m6A in RNA processing, such as RNA decay, translation, splicing, transport and localization.

### The overexpression of YTHDF1, YTHDF3 and KIAA1429 predicts poor survival in BRC patients

**Figure 9:**
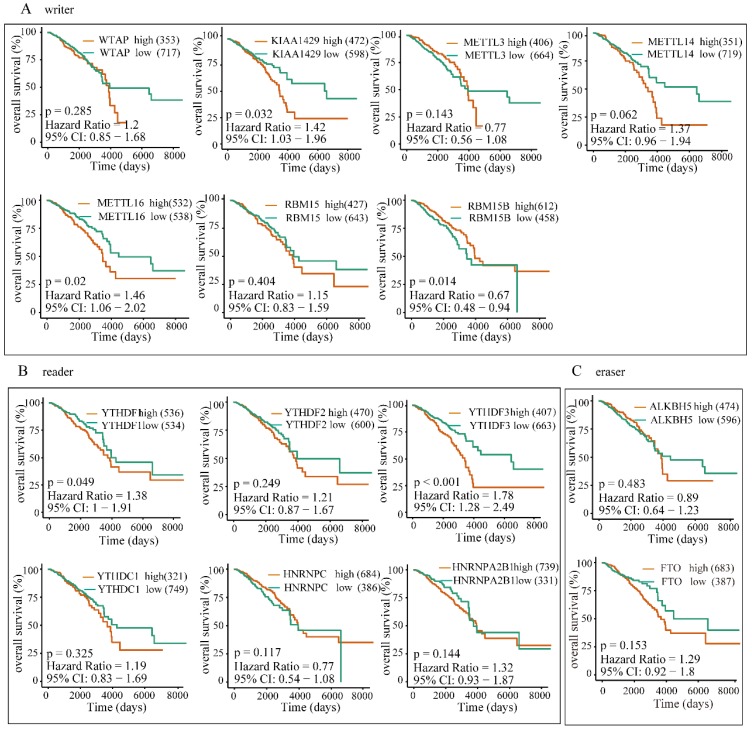
** High expression of YTHDF1, YTHDF3 or KIAA1429 predicts poor prognosis in BRC patients.** The correlations between the expression levels of m6A “writers” **(A)**, “readers” **(B)**, or “erasers”** (C)** and patients OS rates were examined by Kaplan-Meier analysis.

**Figure 10:**
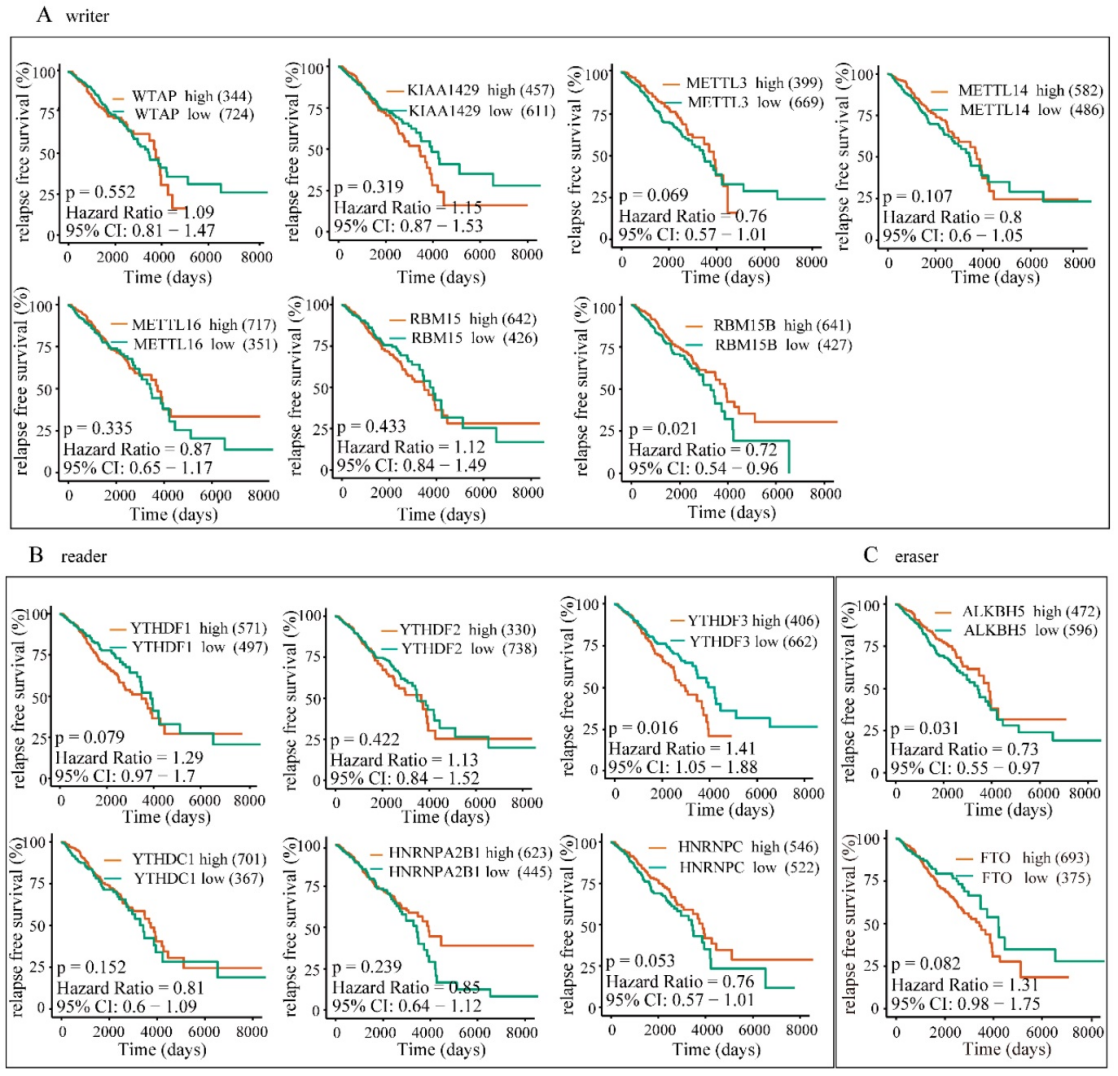
** High expression of YTHDF3 is correlated with lower RFS rates.** The correlations between the expression levels of m6A “writers” **(A)**, “readers” **(B)**, or “erasers”** (C)** and patients RFS rates were examined by Kaplan-Meier analysis.

To further investigate the potential correlation of differentially expressed m6A-related genomic targets to the clinical progression of BRC patients, we analyzed BRC patients' overall survival rates based on the data from TCGA-BRCA cohort. The results demonstrated that increased expressions of KIAA1429 (P=0.032, 95% CI: 1.03-1.96), YTHDF1 (P=0.049, 95% CI: 1-1.91) and YTHDF3 (P<0.001, 95% CI: 1.28-2.49) were significantly associated with poor overall survival rates in patients under the best cut-off value (**Figure 9**). Meanwhile, univariate and multivariate analyses showed that the increased expression of YTHDF3 was an independent predictor of cancer overall survival in patients with BRC (**Table 2**). Furthermore, we also explored whether these m6A-related genomic targets were associated with the relapse-free survival rate of patients. Similarly, we found that overexpression of YTHDF3 was correlated with lower relapse-free survival rate (P=0.016, 95% CI=1.05-1.88) (**Figure 10**).

## Discussion

BRC is characterized by heterogeneity in which genetic or epigenetic factors play indispensable roles in its initiation and progression 17. Currently, early diagnosis and precise individual therapy for BRC remain the greatest challenges. Therefore, to identify consistently altered genomic signatures is critical in BRC basic and clinical research. To uncover new therapeutic targets, we investigated the expression patterns of m6A-related genomic targets in BRC at the mRNA and protein levels. Through transcriptomic and proteomic analyses of m6A-associated genomic targets in large BRC cohorts, including RNA-seq data from public TCGA-BRCA and GEO microarray platforms as well as IHC staining data from the TMA cohorts, we observed that m6A-related genomic targets were frequently dysregulated in BRC and that the upregulation of YTHDF1, YTHDF3 and KIAA1429 is associated with poor patient survival.

**Figure 11:**
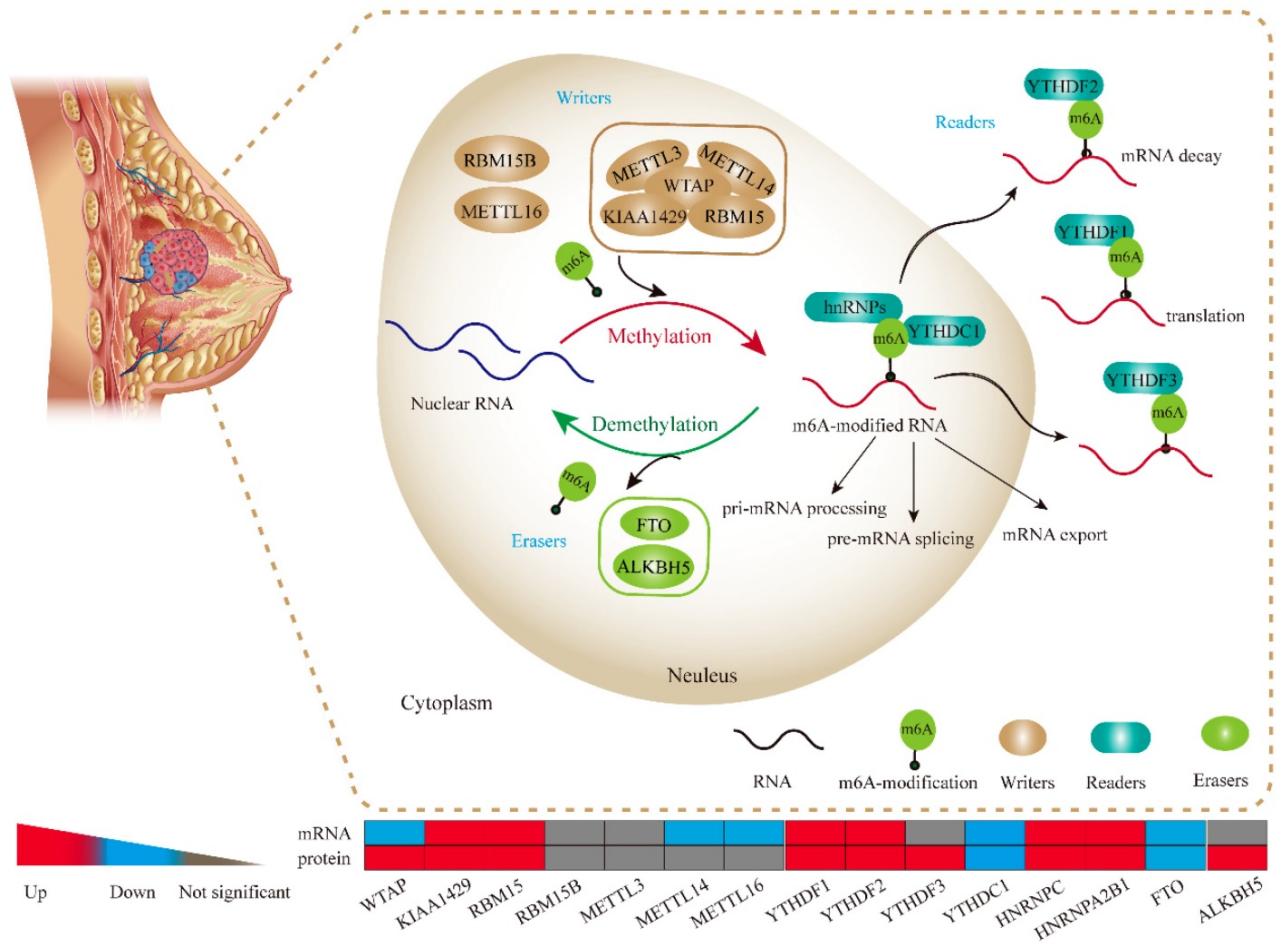
** The potential molecular functions of m6A-based modification of RNA in BRC cell.** m6A RNA methylation, known to be involved in all stages in the processing of RNA, is modulated by its “writers”, “readers” and “erasers”. m6A “writers” mainly refer to methylase complex (WTAP, KIAA1429, RBM15, METTL3 and METTL14), which add m6A modification on RNA, while m6A “readers” could serve as demethylases (FTO and ALKBH5). Meanwhile, both of “writers” and “erasers” dynamically fine-tune the m6A status in RNA within the nucleus. Readers (YTHDF1, YTHDF2, YTHDF3, YTHDC1, hnRNPs) are proteins that bind to m6A modifications and exert various functions including pre-mRNA splicing, pri-miRNA processing, nuclear export, RNA translation modulation and mRNA decay.

As shown in **Figure 11**, m6A is generally considered to be installed by a large methyltransferase complex (m6A 'writers') that adds methylation modifications. WTAP, KIAA1429, METTL3, METTL14 and RBM15 are the core components of this complex 18. Previously, the targetable genomic vulnerability of WTAP has been proposed in a wide spectrum of tumors, including cholangiocarcinoma 19, glioblastoma 20, acute myeloid leukaemia 21 and renal cell carcinoma 22. In line with these studies, we found that WTAP was highly expressed at the protein level in BRC tissues, although it was not consistent with its mRNA level. This finding suggests an important role of WTAP in BRC. METTL3 was shown to participate in hepatocellular carcinoma (HCC) progression via mRNA m6A modification 23. Additionally, RBM15 and METTL14 were demonstrated to play oncogenic roles in leukaemogenesis 24, 25. These observations indicate that m6A dysregulation is a pervasive phenomenon in various malignancies. Similarly, our study observed significant upregulation of RBM15 and KIAA1429 as well as the downregulation of METTL14 and METTL16 in BRC tissues, which suggested that there might also be an abnormal N6-methyladenosine status in BRC.

M6A modification markers can be recognized by proteins, such as DF1, DF2, DF3 and DC1 that contain a specific YTH domain 26. The DF family members (DF1, DF2, and DF3) are highly similar to each other and are predominantly cytoplasmic (**Figure 11**). Two additional members, HNRNPC and HNRNPA2B1, have been identified as nuclear m6A-binding proteins that affect alternative splicing of pre-mRNA and pre-microRNA 15, 27. Previously, YTHDF1 was elucidated to be an oncogene in colorectal cancer 28. YTHDF2 could participate in the progression of HCC and prostate cancer 29, 30. Moreover, HNRNPC was reported to control the aggressiveness of glioblastoma (GBM) cells by regulating PDCD4 31. In support of the role for m6A “readers” in various tumours, we also uncovered a higher abundance of “reader” proteins in BRC tissues. Thus, our study could help further identify m6A reader targets, except for YTHDC1, that are prone to be upregulated in BRC. Intriguingly, another study demonstrated that HNRNPC could contribute to crafting the BRC tumour microenvironment 32. This finding suggests that N6-methyladenosine dysregulation commonly exists and plays a pivotal role in BRC development and progression.

FTO, one of the m6A “erasers” we found to be downregulated in BRC, has been reported to be critical for leukaemogenesis 33. Notably, a previous study revealed that FTO single nucleotide polymorphisms (SNPs) were promising classifiers in predicting BRC risk, which indicates that FTO could function as a novel clinical biomarker 34. Recently, the other demethylase, ALKBH5, was shown to potentiate tumourigenicity of glioblastoma by enhancing FOXM1 expression 35. In our analysis, we also observed a significant up regulation of ALKBH5 at the protein level in BRC. Intriguingly, Zhang et al. identified ALKBH5-mediated modulation of RNA methylation or demethylation involving BRC cell stemness and pluripotency maintenance in a hypoxic environment 36, 37. This evidence strongly supports the fundamental mechanisms of m6A “erasers” in BRC with the induction of demethylation that has been linked to tumour growth and proliferation.

In addition, based on clinical values and on our research, high expression levels of YTHDF1, YTHDF3 and KIAA1429 predicted unfavourable patient prognosis. Moreover, YTHDF3 expression levels are promising independent prognostic factors in BRC, which suggests that YTHDF3 could serve as a novel biomarker for BRC. In agreement with our study, m6A targets already described in the literature were proposed as biomarkers, prognostic indicators or therapeutic targets in cancer. Current research has reported that the elevated expression of YTHDF1 predicts a poor prognosis of HCC 38. Moreover, HNRNPC has been identified as a candidate biomarker in gastric cancer chemoresistance 39. Increased expression of FTO in leukaemia was believed to be associated with sensitivity to the chemotherapy drug R-2HG 40. Therefore, it might be interesting to investigate the role of m6A-related genomic targets in drug resistance mechanisms in BRC. Altogether, in the context of personalized medicine, frequently altered targets could generate detectable mutation patterns in BRC and thus provide a rationale for the advancement of individualized interventions. Furthermore, genome-wide analysis is also a promising prospect for the research of m6A-specific drugs that target unique m6A-related gene patterns. In light of the deregulated m6A gene variability in different patients, exploring an individualized diagnostic and treatment regimen for BRC may be just around the corner.

## Conclusion

M6A-related molecules are frequently dysregulated in BRC and associated with poor patient prognosis. In this study, we provided the first exposition of m6A-related genomic targets in BRC, and the results of our study may open novel avenues for future BRC studies in preclinical and clinical contexts.

## Supplementary Material

Supplementary table.

## Figures and Tables

**Table 1:** GEO Microarray Data enrolled in to Identify Altered m6A Targets in Breast cancer

Accession number	Platform	Number of samples		Country	Years
		Non-tumor	Breast cancer		
GSE70947	Agilent	148	148	USA	2016
GSE15852	Affymetrix	43	43	Malaysia	2009
GSE109169	Affymetrix	25	25	Taiwan	2018
GSE29044	Affymetrix	36	73	Saudi Arabia	2014
GSE24124	Agilent	20	99	Taiwan	2010
GSE36295	Affymetrix	5	45	Saudi Arabia	2014
Total		277	433		

**Table 2:** Independent prognostic factors for OS by multivariate analysis in TCGA BRCA cohort

Risk factors	Clinicopathological features	Univariate analysis			Multivariate analysis		
		HR	95% (CI)	*P* value	HR	95% (CI)	*P* value
Age(years)	≤median	1.000	1.247-2.393	0.001**	1.000	1.350-2.675	<0.001**
	>median	1.727			1.900		
Race	White	1.000	0.611-1.344	0.625			
	Others	0.906					
Her-2	Positive	1.000	0.929-2.108	0.107			
	Negative	1.399					
TNM stage	Stage I and II	1.000	1.950-3.832	<0.001**	1.000	1.941-3.827	<0.001**
	Stage III and IV	2.734			2.725		
KIAA1429	Low	1.000	1.003-1.920	0.048*	1.000	0.553-1.263	0.394
	High	1.388			0.836		
YTHDF1	Low	1.000	1.000-1.920	0.049*	1.000	0.924-1.924	0.124
	High	1.386			1.334		
YTHDF3	Low	1.000	1.299-2.484	<0.001**	1.000	1.031-2.403	0.036*
	High	1.796			1.574		

Abbreviations: CI: confidential interval; HR: hazard ratio; TNM: tumor- node- metastasis. *P < .05, **P < .001.

## Abbreviations

m6A: N6-methyladenosine
BRC: breast cancer
WTAP: Wilms' tumour 1-associated protein
TMA: tissue microarray
TCGA: The Cancer Genome Atlas
GEO: Gene Expression Omnibus
HPA: Human Protein Atlas database
OS: overall survival
RFS: relapse free survival
