# Supplementary material for: N6-methyladenosine-related Genomic Targets are Altered in Breast Cancer Tissue and Associated with Poor Survival
Source: J Cancer. 2019 Aug 29;10(22):5447–59. doi: 10.7150/jca.35053 (PMC6775703; doi:10.7150/jca.35053)
Supplement: Supplementary file 1 — Supplementary table. [file jcav10p5447s1.pdf]

Supplementary Table. Information on antibodies used in this study

	Antibody	IHC	IF	Specificity	Company
**M6A writer**	WTAP	1:500	1:200	Mouse Monoclonal	Proteintech Group, China
	KIAA1429	1:200	1:200	Rabbit Polyclonal	Proteintech Group, China
	RBM15	1:500	1:200	Rabbit Polyclonal	Proteintech Group, China
	RBM15B	1:200	1:200	Rabbit Polyclonal	Proteintech Group, China
	METTL3	1:200	1:200	Rabbit Polyclonal	Proteintech Group, China
	METTL14	1:200	1:200	Rabbit Polyclonal	Proteintech Group, China
	METTL16	1:200	1:200	Rabbit Polyclonal	Proteintech Group, China
**M6A eraser**	ALKBH5	1:500	1:200	Rabbit Polyclonal	Proteintech Group, China
	FTO	1:200	1:200	Rabbit Polyclonal	Proteintech Group, China
**M6A reader**	YTHDC1	1:200	1:200	Rabbit Polyclonal	Proteintech Group, China
	YTHDF1	1:200	1:200	Rabbit Polyclonal	Proteintech Group, China
	YTHDF2	1:200	1:200	Rabbit Polyclonal	Proteintech Group, China
	YTHDF3	1:200	1:200	Rabbit Polyclonal	Proteintech Group, China
	HNRNPA2B1	1:500	1:200	Rabbit Polyclonal	Proteintech Group, China
	HNRNPC	1:200	1:200	Rabbit Polyclonal	Proteintech Group, China
